# *TaAAP6-3B*, a regulator of grain protein content selected during wheat improvement

**DOI:** 10.1186/s12870-018-1280-y

**Published:** 2018-04-23

**Authors:** Xiufeng Jin, Bo Feng, Zhibin Xu, Xiaoli Fan, Jing liu, Qin Liu, Ping Zhu, Tao Wang

**Affiliations:** 1 Chengdu Institute of Biology, Chinese Academy of Sciences, Chengdu, China; 2 University of Chinese Academy of Sciences, NO.19 Yuquan Road, Beijing, China

**Keywords:** GPC, *Triticumaestivum* L., *TaAAP6-3B*, Selection pressure

## Abstract

**Background:**

The content of grain protein (GPC) in cereals is an important part of total protein in human food. Exploring and utilizing new GPC genes is one of the most effective approaches for wheat quality breeding.

**Results:**

Three homoeologues of *TaAAP6(−3A*, *3B*, *3D)*were cloned by homology cloning from *OsAAP6*.Temporal and spatial expression analysis showed that *TaAAP6*homoeologues were preferentially expressed in developing grains, and *TaAAP6-3B* may play a major role in regulating GPC in wheat. Association analysis indicated that*TaAAP6-3B-I* is significantly correlated with higher GPC than that of *TaAAP6-3B-II* for 115 wheat lines in all five environments. *TaAAP6-3B-I,* the favored allele of *TaAAP6-3B,* was preferentially expressed in preliminary developing grain stage. Two functional markers were developed to discriminate 197F_2_populations and the result showed that *TaAAP6-3B-I* (high-protein content) was completely dominant. Two *cis*-regulatory elements appear to be associated with high GPC were found in the 5’UTR of *TaAAP6-3B-I*.The change of the *TaAAP6-3B* locus types indicated that the gene was subjected to selection pressures during long process of artificial selection.

**Conclusions:**

*TaAAP6-3B* is a regulator of GPC and its favored allele *TaAAP6-3B-I* exhibits an obvious potential application in wheat high-GPC breeding.

**Electronic supplementary material:**

The online version of this article (10.1186/s12870-018-1280-y) contains supplementary material, which is available to authorized users.

## Background

Enhancing nutritional quality of cereals is a means of improving human nutrition and health [1]. Nearly one-third of the world population currently suffers from protein malnutrition, and some diseases are caused by poor quality protein and lack of vitamins and other micro-nutrients [2]. The content of grain protein (GPC) in cereals is an important part of total protein in human food. Bread wheat (*Triticum aestivum *L*.*) is one of the most important food crops worldwide, accounting for ~ 20% of all calories and approximately10% of production transforming into protein were consumed [3]. Moreover, the demand for high-quality wheat as a source of protein is expected to increase dramatically in the near future. Cloning GPC-related genes, exploring the favored alleles and developing functional markers could be used in Molecular Module-Based Designer Breeding Systems for wheat.

Common wheat is a hexaploid specie (AABBDD) with a large genome size (17.9 Gb) and abundant repeat sequences (> 80%) [4]. Comparative genomics proved the existence of chromosomes colinearity between wheat and rice. Recently, a few wheat genes were successfully cloned by comparative genomics, such as *TaGW2* [5], *TaGS5* [6], *TaSus1* [7], *TaSus2* [8] et al. Therefore, in combination with completion of a draft for wheat genome sequence, homology-based cloning has become an efficient way to isolate genes in wheat.

GPC is a typical quantitative trait controlled by a complex genetic regulation and influenced by environmental factors and management practices. So far, numerous quantitative trait loci (QTL) associated with GPC have been detected in a number of environments and populations [9–12]. However it is difficult to directly isolate GPC-related genes by map-based cloning strategies due to its huge and complex genome except *Gpc-B1* on 6BS [1], whose identification is significantly meaningful for nutritional quality in cereal crops. Previous studies revealed a putative amino acid transporter *OsAAP6*, which functions as a positive regulator of GPC in rice [3]. As yet, no information is available on the expression of respective homologues of the candidate gene for high contents of grain protein in common wheat. Therefore, our objectives were to clone homologues of *TaAAP6*, and analyze the temporal and spatial expression of them to confirm candidate gene. Association analyzes of GPC haplotypes alleles for 115 wheat cultivars in all five environments. Evaluate potential *cis*-elements associated with GPC diversity. And then, in order to understand the selection intensity of candidate gene block in manual selective breeding, the high-density wheat 90 K Illuminai Select SNP array [13] were selected to find polymorphism of the locus block.

## Results

### *TaAAP6-3B*、*3D* show high identity to *OsAAP6*

**Fig. 1:**
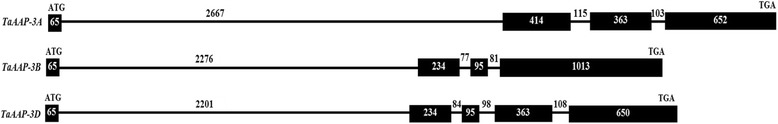
Gene structure of three *TaAAP6 *homoeologues in Chinese Spring. Solid blocks indicate exons; lines between exons represent introns. Numbers under exons and introns denote size (bp)

Based on the conserved sequences of *OsAAP6* (NCBI Accession number:KM213630), *TaAAP6* genomic and CDS were blast from wheat genome in Chinese Spring, and analytical scorings were likely to located on 3A、3B、3D chromosome. Their genomic sequence lengths were 4379, 3841 and 3898 bp, encoding putative 492, 467 and 467 amino acids respectively. Full-length cDNA of the genes were amplified by 5’-UTR and 3’-UTR genome-specific primers, *AFc*, *BFc*, *DFc* (Additional file 1: Table S2). Compare to four exons in *OsAAP6*, *TaAAP6-3A* and *TaAAP6-3B* consisted of four exons, and *TaAAP6-3D* consisted of five exons (Fig. 1). All of them contain a large first intron, which is similar to that of *OsAAP6*.

The cluster analysis of *TaAAP6* patterns for the three genes showed that the similarities between them are up to 75.90%. Based on the amino acids sequences and domains, pair wise comparison result showed that *TaAAP6-3B* - *TaAAP6-3D* shared a higher identity (97.86%) than that of *TaAAP6-3A* - *TaAAP6-3D *(57.40%) and *TaAAP6-3A* - *TaAAP6-3B* (58.42%). Moreover, *TaAAP6-3A* contained five insertions, and there are only six different amino acid residues between *TaAAP6-3B* and *TaAAP6-3D.* Sequence alignment result shows that *TaAAP6-3B* and *TaAAP6-3D* were more similar to *OsAAP6* than*TaAAP6-3A* (Additional file 2: Figure S1).

### Phylogenetic analyses

**Fig. 2:**
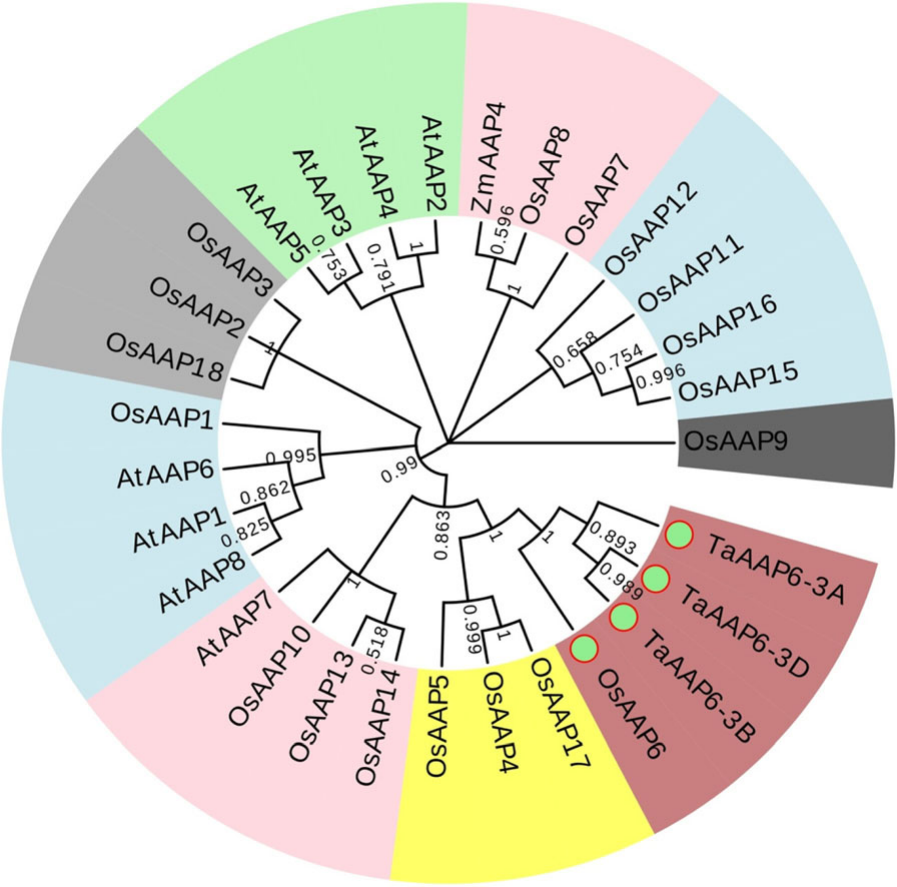
Phylogenetic analyses of the AAPs**.** At (*Arabidopsis thaliana*), Os (*Oryza sativa*) and Zm (*Zea mays*). The genes are as follows: *AtAPP1*(Gene ID: At1g58360), *AtAPP2* (Gene ID: At5g09220), *AtAPP3*(Gene ID: At1g77380), *AtAPP4*(Gene ID: At5g63850), *AtAPP5*(Gene ID: At1g44100), *AtAPP6*(Gene ID: At5g49630), *AtAPP7*(Gene ID: At5g23810), *AtAPP8*(Gene ID: At1g10010), *OsAPP1*(Gene ID: LOC_Os07g04180), *OsAPP2*(Gene ID: LOC_Os06g12330), *OsAPP3*(Gene ID: LOC_Os06g36180), *OsAPP4*(Gene ID: LOC_Os12g09300), *OsAPP5*(Gene ID: LOC_Os01g65660), *OsAPP6*(Gene ID: LOC_Os01g65670), *OsAPP7*(Gene ID: LOC_Os05g34980), *OsAPP8*(Gene ID: LOC_Os01g66010), *OsAPP9* (Gene ID: LOC_Os02g01210), *OsAPP10*(Gene ID: LOC_Os02g49060), *OsAPP11*(Gene ID: LOC_Os11g09020), *OsAPP12*(Gene ID: LOC_Os12g09320), *OsAPP13*(Gene ID: LOC_Os04g39489), *OsAPP14*(Gene ID: LOC_Os04g56470), *OsAPP15*(Gene ID: LOC_Os12g08130), *OsAPP16*(Gene ID: LOC_Os12g08090), *OsAPP17*(Gene ID: LOC_Os06g12350), *OsAPP18*(Gene ID: LOC_Os06g36210), *ZmAPP4*(Gene ID: GRMZM2G110195). The phylogenetic tree is produced by online software (http://www.evolgenius.info/evolview/#login) based on the comparison of amino acid sequences

Amino acid transporters/permeases are key regulators of plant metabolism, growth and development [14]. A phylogenetic analysis was performed by generating a neighbor-joining phylogenetic tree (Fig. 2), consist of one member (*ZmAAP4*) in maize, eight members (*AtAAP1–8*) in Arabidopsis,18 members (*OsAAP1–18*) in rice (http://aramemnon.botanik.uni-koeln.de/). Five clusters were formed according to differences in amino acid sequences of AAPs, both *TaAAP6* and *OsAAP6* are classed as a subgroup, which means *TaAAP6* may be the ortholog of *OsAAP6*, and *TaAAP6-3B* was closer to *TaAAP6-3D* than to *TaAAP6-3A* (Fig. 2). Previously, the *TaAAP* family have been mapped to wheat chromosome and it has been named as *TaAAP8* [15].

### *TaAAP6-3B* was preferentially expressed in developing grains

**Fig. 3:**
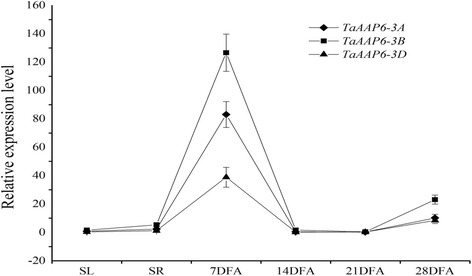
The comparative expression pattern of *TaAAP6 *homoeologues. SL, seedling leaf; SR, seedling root; various stages of grain development, including 7DAF, 14DAF, 21DAF, 28DAF (days after flowering). Error bars denote ±SD

In order to investigate the temporal and spatial expression patterns of the *TaAAP6 *homoeologues in different tissues and organs, specific primers *qA*, *qB* and *qD* (Additional file 1: Table S2) were used for quantitative real-time PCR (qRT-PCR). The three homoeologues were ubiquitously expressed with similar patterns in various tissues, but showed higher expression in developing grains than in established roots and seedlings (Fig. 3). The most abundant expression was at 7 days after flowering (DAF) in endosperm, then gradually declined until 21 DAF, and increased at 28 DAF. This result showed a similar “high-low-high” dynamic trend to the protein content change during grain filling stage [16]. Although three homologous genes showed similar expression patterns, they had significantly different expression abundances. Compared to *TaAAP6-3A* and *TaAAP6-3D*, *TaAAP6-3B* was expressed at a much higher level, one and half timesto that of *TaAAP6-3A* and three times to that of *TaAAP6-3D* (Fig. 3).

### Allelic variations of *TaAAP6-3B*

**Fig. 4:**
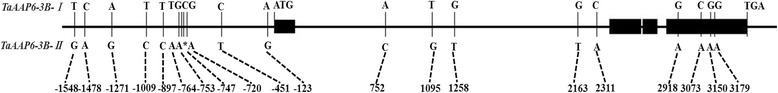
Gene structure and natural variation of the two *TaAAP6-3B* haplotypes

To detect sequence variations of *TaAAP6-3B*, three pairs of primers *BF1*, *BF2* and *BF3* were designed to amplify the genome-specific fragments, and full-length sequence of *TaAAP6-3B* gene was assembled. Based on the nucleotide polymorphisms identified among the 115 Chinese wheat accessions (Additional file 3: Table S1), the sequences were divided into two haplotypes, which were named *TaAAP6-3B-I* and *TaAAP6-3B-II*. 17 synonymous mutation sites were identified in genomic sequence between them, and the coding regions contained 4 synonymous mutation sites (Fig. 4). Meanwhile, ten sites were detected in the promoter region, and a nucleotide deletion was found in *TaAAP6-3B-II*.

### Two *Cis*-acting elements associated with GPC were found in *TaAAP6-3B-I*

Previous studies have shown that, *Cis*-regulatory elements (CREs) can impact gene expression levels, but also developmental timing and tissue specificity of expression [17]. In rice, *OsAAP6* expression was regulated by three CREs, including Sulphur-responsive element, copper-response element and inr-element in high GPC population [3]. In *TaAAP6-3B*, promoter region (~ 1.8 kb) was amplified by specific primers *BF1* and *BF2* (Additional file 1: Table S2). The result shows that six CREs in upstream sequence were identified in *TaAAP6-3B-I* and not detected in*TaAAP6-3B-II*(Additional file 4: Table S4). Two CREs target for transcriptional activators and regulators may be associated with high GPC for them. One (− 753 bp) is a Sulfur-responsive element [18, 19], another (~ 1009 bp) is a SEF4 motif which interacts with a soybean storage protein enhancer [20]. Taken together, our results imply that the two common variations in the two potential CREs of the *TaAAP6-3B* 5’-UTR appear to be associated with GPC diversity in the populations.

### *TaAAP6-3B- I* shows a much higher expressing level

In order to investigate the difference expression patterns of the two haplotypes, two primers *Actin1*, *qB* (Additional file 5: Figure S2) were used to detect the expression patterns through qRT-PCR in modern cultivars. *TaAAP6-3B-I* and *TaAAP6-3B-II* was expressed with similar pattern in six tissues. However, they had significantly different expression abundances. Compared to *TaAAP6-3B-II*, *TaAAP6-3B-I *was expressed at a much higher level in every stage,especially in the endosperm at 7 DAF, the expression excelled two times (Additional file 5: Figure S2).

### *TaAAP6-3B-I *associates with high GPC in natural and genetic populations

**Fig. 5:**
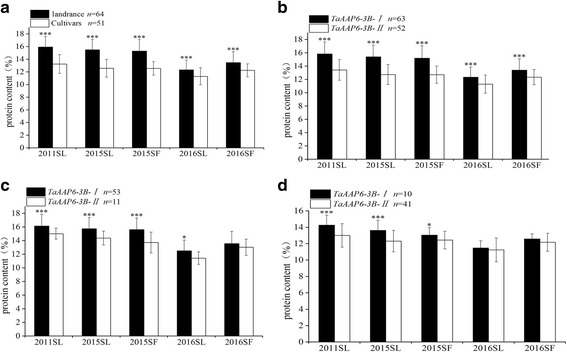
Correlation analyses of grain protein contents (GPC) in 115 lines. **a** The GPC between landraces and modern cultivars; **b** The GPC between *TaAAP6-3B-I* and *TaAAP6-3B-II*; **c** The GPC between two haplotypes in landraces; **d** The GPC between two haplotypes in modern cultivars. n, is the number of accessions. Significant differences at *P = 0.05, **P = 0.01and***P = 0.001, respectively. Error bars denote±SD

The content of grain protein (GPC) was measured in 115 hexaploid wheat lines. Compared to 51 modern cultivars, the 64 landraces exhibited a significantly higher GPC in all five environments (*P* < 0.001) (Fig. 5a) and the phenotypic differences between the two lines were 20.31, 23.33, 21.66, 9.04, and 9.96(%), respectively (Additional file 6: Table S3).

After genotyping, association analysis between phenotypes and genotypes was performed. The results show that the GPC of *TaAAP6-3B-I* issignificantly higher thanthat of *TaAAP6-3B-II* (*P* < 0.001) (Fig. 5b), and differences between the two haplotypes were 17.94, 20.85, 19.58, 9.29 and 8.53 (%) in all five environments, respectively (Additional file 6: Table S3). Furthermore, in landraces, the significant differences between 53 *TaAAP6-3B-I* lines and 11 *TaAAP6-3B-II* lines were detected in four environments (Fig. 5c). The mean differences between the two alleles were 7.50, 9.55, 13.77, and 9.37 (%), respectively (Additional file 6: Table S3). For modern cultivars, the two haplotypes detected in three environments have significant differences (Fig. 5d). The mean differences were 9.66, 10.69 and 4.87(%), respectively (Additional file 6: Table S3).

**Fig. 6:**
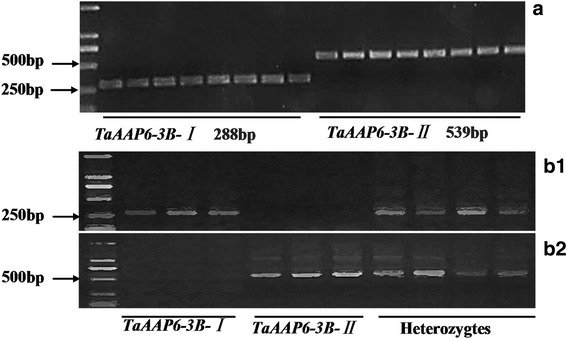
Two markers developed for identify two haplotypes of *TaAAP6-3B*. (**a**) PCR products of *TaAAP6-3B* in natural populations; (**b1**), (**b2**) PCR products of *TaAAP6-3B* in F_2_ populations

To further distinguish the twohaplotypes of *TaAAP6-3B*, two pairs of complementary primers (Additional file 1: Table S2) were designed. The PCR product of *TaAAP6-3B-I* amplified by the genomic-specific primer pairs *BI* was 288 bp, whereas that of *TaAAP6-3B-II *has not emerged. And for *TaAAP6-3B-II*, 539 bp band could be amplified with primer pairs *BII*, whereas that of *TaAAP6-3B-I* has not emerged (Fig. 6a).

The two markers were also used for detecting genetic populations (Fig. 6 b1, b2). Analysis of a F_2_ population containing 197 individuals derived by ZM5453 (*TaAAP6-3B-I*) × ZhongKemai138 (*TaAAP6-3B-II*) showed that GPC in heterozygous plants was significantly lower than that in *TaAAP6-3B-I* homozygotes but higher than that in *TaAAP6-3B-II* homozygotes (Additional file 7: Table S5), which indicates that the high-protein content allele *TaAAP6-3B-I* was completely dominant.

### Changing of the locus polymorphism during wheat improvement

**Fig. 7:**
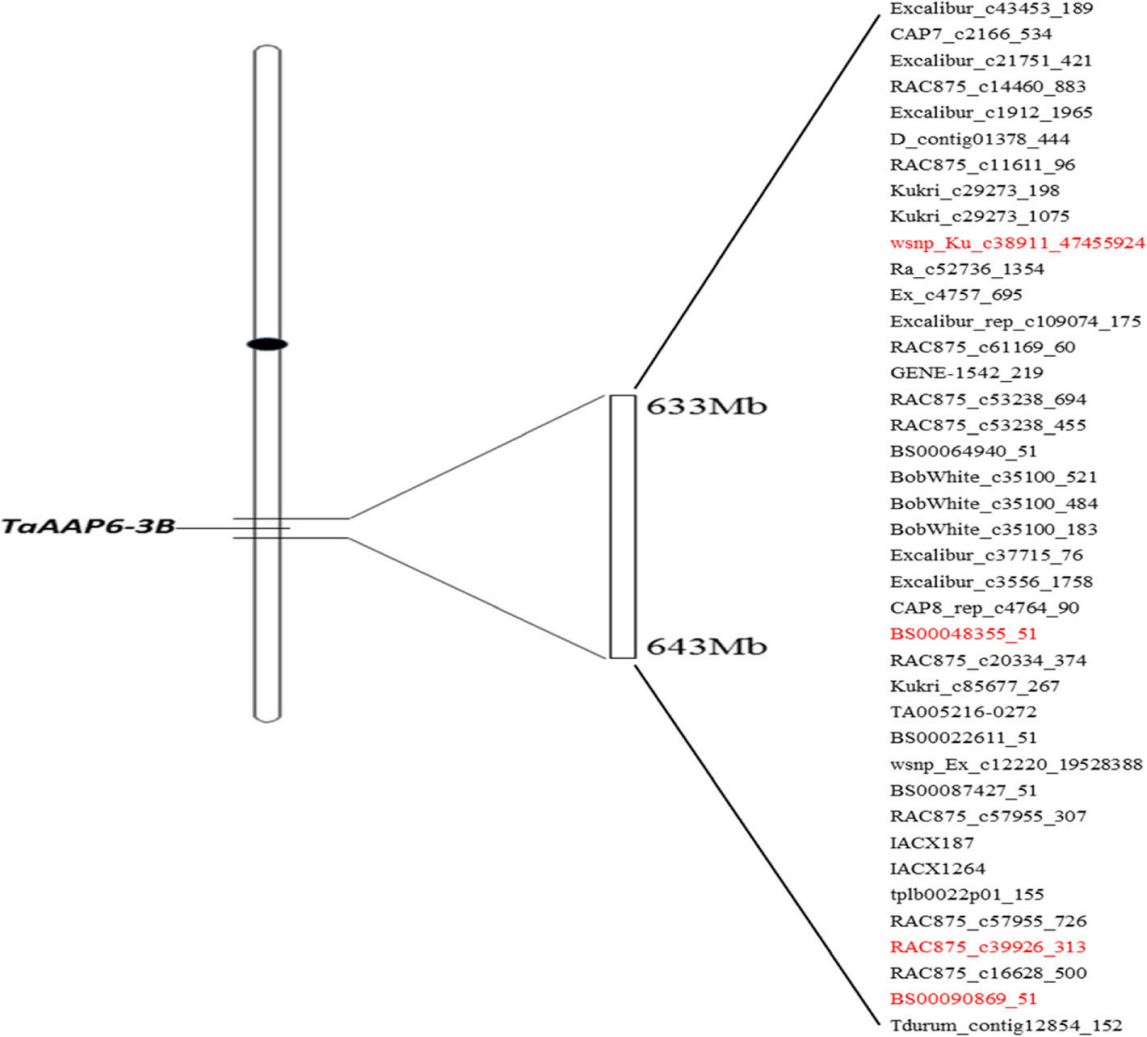
40 SNPs in the *TaAAP6-3B* locus on 3B chromosome. The reds are the polymorphic SNPs in 115 lines

Domestication and modern plant breeding have previously narrowed the genetic base of bread wheat. The detection of loci under selection during crop improvement can contribute to more targeted breeding efforts and the opportunity to improve genomic selection models [21]. In order to detect the variation of the locus polymorphism during artificial selection, 40 high-density SNPs around the *TaAAP6-3B* locus on 3B chromosome was used for genotyping in 115 wheat lines, and four polymorphic SNPs were found (Fig. 7).

**Fig. 8:**
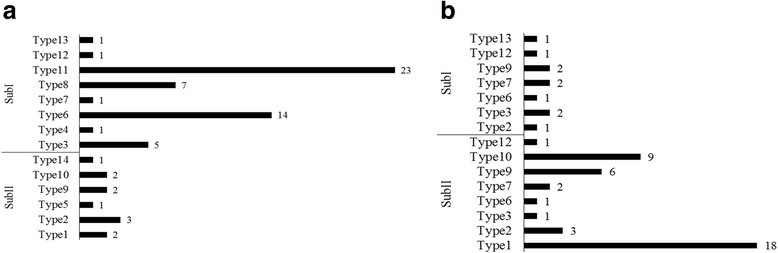
Types of the *TaAAP6-3B* locus in modern cultivars and landraces. **a**: landraces; **b**: cultivars. SubI: group of *TaAAP6-3B-I*, SubII: group of *TaAAP6-3B-II*

For wsnp_Ku_c38911_47455924, three variations AA, AG, GG were found. And two variations GG, AG; GG, AG and TC, CC were detected at BS00048355_51;RAC875_c39926_313 andBS00090869_51, respectively.These four SNPs constitute 24 types of the locus polymorphism in theory; nevertheless only 14 types were found in our study (Additional file 6: Table S3). With all 14 types among landraces, type 11 constituted 35.3%; and type 6 (23.5%); type 8 (11.8%); type 3 (7.4%); type 2 (5.9%) and the remaining under 5%, respectively. Of all 9 typesin cultivars, type 1 constituted 37.5%, and type 10 (20.8%); type 9 (18.8%); type 5 (10.4%); type 2 (6.3%) and the remaining under 5%, respectively(Fig. 8).

Meanwhile, five locus types (4, 5, 8, 11, 14) in landraces have been selected off, of which type 8 and 11 account for 70.6% lines among SubI. Interestingly, three types (1, 9 and 10) account for only 8.8% lines in landraces, but account for 64.6% lines in SubII for cultivars. The above results show that, after artificial selection, the SubI (high GPC populations) were gradually replaced by SubII (low GPC populations) (Fig. 8).

## Discussion

### *TaAAP6-3B* regulates GPC similar to *OsAAP6*

Wheat GPC is a multigenic-controlled complex trait, and the demand for high-quality cereals as a source of protein has become increased [15]. So far, only *NAM1* gene has been reported to enhance the nutritional value of crops, which was associated with increased GPC in bread wheat [1]. Efforts to identify more genes affected GPC and direct selection of the alleles with positive effects is necessary.

Physiological data further suggest that amino acid permeases are key regulators in plant metabolism and that their activities affect growth and development [16, 17]. In Arabidopsis, *AtAAP1* and *AtAAP5* are important to uptake of neutral and acidic amino acids [18]; *AtAAP2* to sinks is decreased leading to reduce total nitrogen and protein levels in seeds [22]. *AtAAP8* mediates amino acid uptake into the endosperm at the early embryo stage [20]. In rice, *OsAAP8* and *OsAAP15* might participate in the uptake and long-distance transport of amino acid [23], *OsAAP6* has pleiotropic effects on grain storage materials.

Our study showed that *TaAAP6* is a member of AAP family genes (Fig. 2), and very closer to *OsAAP6*. QRT-PCR results show that *TaAAP6-3B* has the highest express level, which suggests it may be the important homologue. Allelic variations in 115 wheat lines contained two haplotypes *TaAAP6-3B-I* and *TaAAP6-3B-II*. The expression pattern at stages of seed development showed *TaAAP6-3B-I* in developing seeds were significantly positively associated with higher GPC than *TaAAP6-3B-II* (Additional file 5: Figure S2). Association analysis showed that *TaAAP6-3B-I* was a favored allele associated with higher GPC both in natural and genetic populations.

*Cis*-Regulatory elements (CREs) control the expression of an unmodified coding sequence during artificial selection in crops. In-depth understanding of gene regulatory networks and genome editing to find and alter CREs at the single nucleotide level in plant genomes may provide a promising engineering strategy for future crop improvement [24]. In rice, three CREs, Sulphur-responsive element, copper-response element and inr-element are key factors to be associated with GPC diversity [3]. Our results showed that two CREs may play important roles in controlling wheat GPC by regulating the *TaAAP6-3B* expression. One is sulphur-responsive element, the binding sequence of auxin response factor, which targets for transcriptional activators and involved in a broad range of responses [18, 19]. And another is SEF4 motif, which was found to increase in soybean embryos during the time of β-conglycinin synthesis [20].

### *TaAAP6-3B-I* has subjected to strong selection pressure in wheat improvement

Wheat (*Triticum aestivum* L.) is one of the founder crops that likely drove the Neolithic transition to sedentary agrarian societies in the Fertile Crescent more than 10,000 years ago [25]. During the past centuries, so many traditional landraces were continually replaced by modern cultivars to adapt to specific conditions of human requirements [26]. The genetic variation was subsequently reduced by selection especially for genes controlling agronomic traits, such as TKW and GPC.

**Fig. 9:**
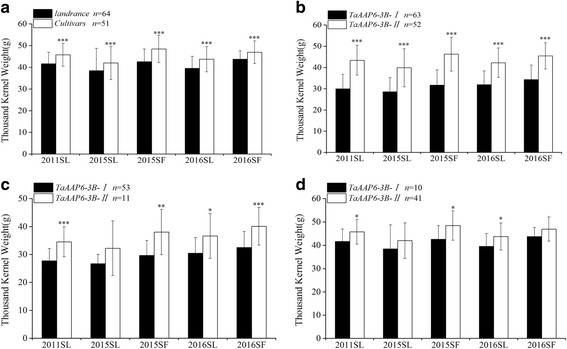
Correlation analyses of thousand-kernel weight (TKW) in 115 lines. **a** The TKW between landraces and modern cultivars; **b** TKW between phenotypic of *TaAAP6-3B-I* and *TaAAP6-3B-II*; **c** The TKW between two haplotypes in landraces; **d** The TKW between two haplotypes in modern cultivars. n, is the number of accessions. Significant differences at *P = 0.05, **P = 0.01 and ***P = 0.001, respectively. Error bars denote±sd

Previous results suggest a negative relationship between TKW and GPC [27, 28]. Grain yield, the primary breeding objective, has been substantially increased during selection. TKW, a major component of yield, has translated into continuous genetic gains [29]. In this study, we compared the TKW of *TaAAP6-3B-I* and *TaAAP6-3B-II* in modern cultivars and landraces at all five environments (Additional file 1: Table S2). The result also revealed the negatively relationship between TKW and GPC (Figs. 5 and 9). This means in the long-term selection of breeding, the GPC gradually decreased, while the TKW gradually increased. It was also confirmed that both traits are the products of complex interdependencies between plant developmental traits and yield components. In our 197 F_2_ genetic populations, the number of *TaAAP6-3B-I* homozygotes is two times of *TaAAP6-3B-II* homozygotes. This phenomenon could be explained due to the physical interval associated with segregation distortion loci [30, 31]. Although the ration of *TaAAP6-3B-I* is much higher in a segregation population (41.62%) (Additional file 7: Table S5), its ration in modern varieties is lower (19.61%) (Fig. 5d). It suggests that *TaAAP6-3B-I* haplotype was selected off during artificial selection. The similar results can be found in changing of *TaAAP6-3B* haplotype block types. In landraces, *TaAAP6-3B-I* associate with high GPC and low TKW is dominant(82.81%). In modern varieties, *TaAAP6-3B-II* associate with low GPC and high TKW is dominant (82.00%). As we know, the main objective of breeding is focus on yield, and TKW is under a positive selection. Because of the negative relationship of GPC and TKW, GPC may under a negative selection. As a result, all these findings indicate that *TaAAP6-3B* gene has subjected to strong selection pressure in wheat improvement.

### The functional markers for the future

It is necessary to use gene-specific markers for selection high GPC in wheat breeding. In this study, two complementary pairs of functional markers were developed to distinguish the two haplotypes of *TaAAP6-3B*. Lines carrying *TaAAP6-3B-I* exhibited significantly higher GPC than those carrying *TaAAP6-3B-II* in both established natural and genetic populations (Fig. 5). As developments of other favored allele’s functional markers, containing quality traits, agronomic traits and disease resistance, the two functional markers could be used in Molecular Module-Based Designer Breeding Systems for wheat.

## Conclusions

In conclusion, *TaAAP6-3B,* the ortholog of *OsAAP6,* was cloned and analyzed. The expression patterns, haplotypes, *cis*-regulatory elements and locus polymorphism were conducted to underlie its effect to GPC. *TaAAP6-3B-I,* the favored haplotype of *TaAAP6-3B,* is associated with significantly higher GPC than that of*TaAAP6-3B-II*. Meanwhile, this haplotype has undergone the strong selection pressure in artificial selection during wheat breeding. This study provides important genes and functional markers for wheat breeding in nutritional quality improvement.

## Methods

### Plant material and growth condition

A total of 115 bread wheat lines, collected from our lab (Pro Tao Wang of Chengdu Institute of Biology, Chinese Academy of Sciences) were used for this study, including 64 landraces and 51 modern cultivars. Wheat plants were grown in two locations, Shuangliu (SL) (103.87°E, 30.59°N) and Shifang (SF) (104.01°E, 31.29°N), in Sichuan Province by three growing seasons, 2010–2011, 2014–2015 and 2015–2016. In addition, a segregation population contains 197 F_2_ was grown in 2016–2017 growing season at Shuangliu. The lines were sown in single row of 1.2 m long and 0.2 m apart with three replications per site. Fields were managed according to local agricultural practices.

### Measurement of GPC and TKW

After harvested, three plants of each line per replication were selected at random for phenotype analysis. Fully filled grains were used for measuring content of grain protein (GPC) and determined twice for each line by a Kjeltec 2200 system (FOSS). And, three independent samples of 200 grains were converted to one thousand-kernel weight (TKW) by SC-E software (Hangzhou Wanshen Detection Technology Co., Ltd., Hangzhou, China).

### DNA and RNA extraction

Genomic DNA from all plant materials was extracted from young leaves by the CTAB method. Total RNA was extracted from various plant tissues with an RNA extraction kit and TRIzol reagent (Invitrogen). First-strand cDNA was synthesized by 2 mg RNA and 200 U M-MLV reverse transcriptase (Promega) in 20ul volume, and subsequent for quantitative real-time PCR (qRT-PCR).

### Primers and PCR amplification

All primers used in this study were designed by Primer Premier 5.0 software, are listed in (Additional file 1: Table S2), and synthesized in Chengdu TSINGKE Biological Technology Co., Ltd. PCR was performed in total volumes of 20 uL, including 50 ng DNA, 0.5uL 10 mM forward and reverse primers, 2 uL of 25 mM dNTP, 2 uL GC buffer and 0.45 uL KOD Plus polymerase (TOYOBO, Shanghai, China). PCR were performed as follows: 94 °C for 4 min; followed by 35 cycles of 94 °C for 35 s, annealing for 35 s and 72 °C for extension (30 s–2 min); and 72 °C for 10 min with a final extension. The annealing temperature and extension time depended on the primer sets and the length of PCR products. All PCR products were separated via gel-electrophoresis on a 1.5% agarose-gel stained with ethidium bromide and visualized by UV light. To examine the accuracy of primers, PCR products were selected for DNA sequencing and analysis.

### Isolate *TaAAP6-3A, 3B and 3D* in wheat

The homologous sequence of *OsAAP6* in wheat was obtained via blast in NCBI. Chinese Spring was used to clone the sequence of *TaAAP6*. Ninepairs of Primers *AF1*, *AF2*, *AF3*, *BF1*, *BF2*, *BF3*, *DF1*, *DF2*, *and DF3* were designed to amplify the Genome-specific fragments according to the blast sequence, and full length sequence of *TaAAP6-3A*, *TaAAP6-3B, and TaAAP6-3D* gene was assembled with the three fragments performed by DNAMAN. The PCR products werecloned into the pEASY-Blunt simple vector and transformed to DH5α competent *E. coli* cells by the heat shock method (Transgen, Beijing, China, Product Code: CT101). Positive clones were selected for sequencing (TSINGKE, Chengdu, China). PCR and positive clones were repeated at least three times.

### Phylogenetic and *Cis*-acting elements analysis

Phylogenetic trees were constructed based on the full-length amino acid sequences of *TaAAP6-3B*, and other AAP6s in plants. Phylogenetic analysis was performed by MEGA7.0 software, and using multiple algorithms (neighbor joining, minimum evolution, maximum likelihood, Nei and Kumar 2000) and different distance model (P versus PC distance) and gap options (complete versus pairwise deletion). Bootstrap values were estimated based on 1000 replications.

The core elements of the promoters were identified using the TSSP program. *Cis*-acting regulatory elements of the promoter were predicted by PLACE analysis.

### *TaAAP6-3B *haplotypes and functional markers development

In order to test the genotype of *TaAAP6-3B* alleles, three pairs of Primers *BF1*, *BF2*, *and BF3* were used to clone the sequence in 115 lines (Fig. 2). SNPs were identified using DNA Star software. Two markers (*BI*, *BII*) were designed by haplotypes to distinguish cultivars and F_2_ populations, respectively. Genome-specific fragments were amplified by the corresponding primers and then separated by electrophoresis in agarose-gel. To examine the accuracy of functional markers, PCR products were selected for DNA sequencing and analysis.

### Expression analysis

Quantitative real-time PCR was carried out in a total volume of 20 ul containing 0.5 ul of the reverse-transcribed product, 0.4 ul of genes specific primers *qA*, *qB*, *qD* (Additional file 1: Table S2), 0.4 ul Passive Reference DyeII, 10 ul of 2 × Trans Start Top Green qpcrSuperMix (Transgen, Beijing, China) on an ABI 7500 real-time PCR system (Applied Biosystems, USA), according to the manufacturer^’^s instructions. A sequence about 170 bp was amplified as a reference gene to continue. Measurements were obtained by means of the relative quantification method [32]. All expression level data obtained by quantitative real-time PCR are based on at least four biological samples on which three replications of the technique were conducted.

### *TaAAP6-3B* locus polymorphism

The size of 3B chromosome contents ~ 740 Mb, and 79 locus blocks [30], which means the average size of one block contains ~ 10 Mb. As a result, each ~ 5 Mb upstream and downstream the *TaAAP6-3B* was selected from the wheat genome to analysis the Locus polymorphism. 40 SNPs were found in this region from the high-density wheat 90 K Illumina iSelect array in 3B chromosome. These SNPs were used for genotyping in 115 wheat lines at the Compass Biotechnology Co., Ltd.

### Statistical analyses

To determine phenotypic differences between genotypes, all data obtained were subjected to ANOVA, and the mean difference was compared by the LSD test at 95% levels of probability. Sequences were analyzed by DNAMAN software. In all figures, the spread of values is shown as error bars representing standard errors of the means.

## Additional files

Additional file 1: **Table S2.** Primers for cloning and functional analysis of *TaAAP6-3B*. (DOCX 15 kb)

Additional file 2: **Figure S1.** Sequence alignment of *OSAAP6* and three *TaAAP6* homoeologues. (PPTX 795 kb)

Additional file 3: **Table S1.**TaAAP6-3B genotypes and Locus polymorphism in 115 wheat lines. (DOCX 31 kb)

Additional file 4: **Table S4.** Putative *cis*-regulatory elements detected in *TaAAP6-3B-I*. (DOCX 15 kb)

Additional file 5: **Figure S2.** Comparative expression pattern of *TaAAP6*-3B. (PPTX 77 kb)

Additional file 6: **Table S3.** Comparisons the GPC and TKW between *TaAAP6-3B-I* and *TaAAP6-3B-II* about 115 lines in five environments. (DOCX 18 kb)

Additional file 7: **Table S5.** Contents of protein grain from genotypic classes among 197 individuals from a F_2_ population. (DOCX 15 kb)

## Abbreviations

ANOVA: Analysis of variance
CREs: *Cis*-regulatory elements
DAF: Days after flowering
GPC: Content of grain protein
NCBI: National Center for Biotechnology Information
QTL: Quantitative trait loci
TKW: Thousand kernel weight
