# Supplementary material for: TaAAP6-3B, a regulator of grain protein content selected during wheat improvement
Source: BMC Plant Biol. 2018 Apr 23;18:71. doi: 10.1186/s12870-018-1280-y (PMC5914022; doi:10.1186/s12870-018-1280-y)
Supplement: Supplementary file 1 — Table S2. Primers for cloning and functional analysis of TaAAP6-3B. (DOCX 15 kb) [file 12870_2018_1280_MOESM1_ESM.docx]

**Table S2**

| Name | Forward primer (5'-3') | Reverse primer (5'-3') |
| --- | --- | --- |
| AF1 | CTGGGAAAGGAAGCGAGGGACA | CGCTATCTACCTCGCTCCCTACTG |
| AF2 | CCGTGACGCAAATACTGGCAAAC | TTTGTTGTTACCGAGGCAGGA |
| AF3 | GTCCGCTCCTGCCTCGGTAAC | GCTGATTATTTGACACTTCCACC |
| BF1 | GGCTACAATACCCTAACTTTCC | TACAAGTCACATAAGTACCGACA |
| BF2 | CGTTGGCGTCAAGGTCAGA | CGGTGTTAGGATTTAGGAGGA |
| BF3 | AGTATCAGAATCCCGCAAAG | GCTACAAATCGGCTAAACAG |
| DF1 | GCCTGTGAATCCATCTCCC | CGGTGTATCTGGACGCTGT |
| DF2 | CCAACAGCGTCCAGATACAC | AGGTCCTTGCTTTCCGATTA |
| DF3 | CCTTCAACTTGTGCTCCCTC | CCAGCAACCAGCTATACCAA |
| AFc | AAGAGGCGGGGAGAGGGAAAG | GCAGCAGCCACCTCCATATAG |
| BFc | CGGTGCCCGAGCGGAGGAAGAG | AAACAGAGCACACACGGAACAT |
| DFc | GGGAGAGAGAGACAGAGAGAGAGAT | TCATCAGAGCAGCCACCTCCATA |
| BI | CGGAAATGATTTTTGCACCT | GCATGGTAATACGTGTCTCA |
| BII | GCGCGTCGGCTACTCATGAT | GCATGGTAATACTGTCTTATT |
| ACTIN | TACTCCCTCACAACAACCG | AGAACCTCCACTGAGAACAA |
| qA | GCTCGGCATTCACTCGTTTCCTTG | CACCTGACCGGCCTTCCACTG |
| qB | TCGGCATTCACTCGTTTCCTTG | CTTTTAATGCGGCGATCGGTCAAT |
| qD | GATTCGTGGGATTCCGTGAGGC | TTCTTTTAATGCGGCGATCGG |
