# Supplementary figures and images for: TaAAP6-3B, a regulator of grain protein content selected during wheat improvement
Source: BMC Plant Biol. 2018 Apr 23;18:71. doi: 10.1186/s12870-018-1280-y (PMC5914022; doi:10.1186/s12870-018-1280-y)

## Slide 1
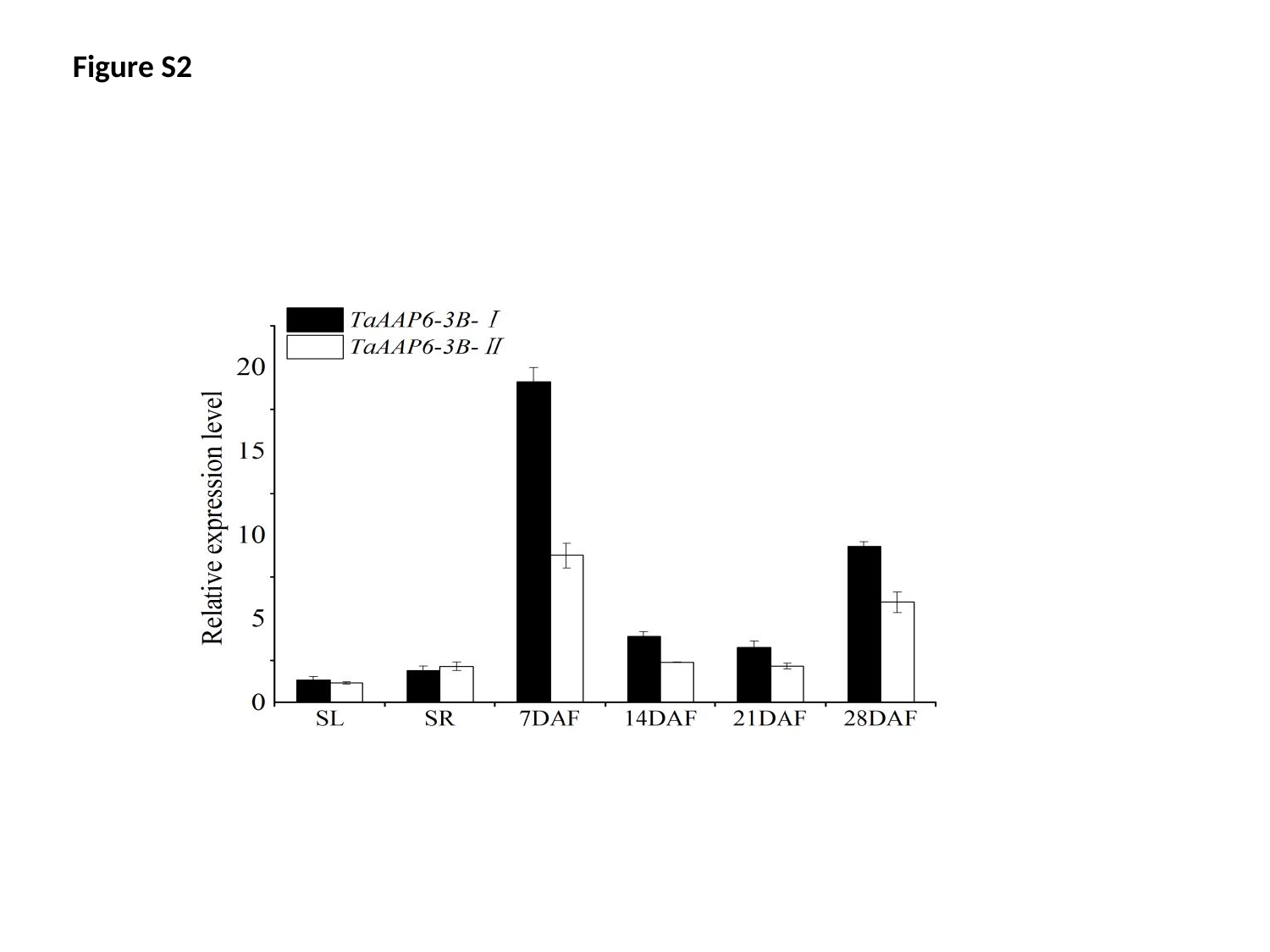

Figure S2

Supplement: Supplementary file 2 — Figure S1. Sequence alignment of OSAAP6 and three TaAAP6 homoeologues. (PPTX 795 kb) [file 12870_2018_1280_MOESM2_ESM.pptx]
