# Supplementary material for: TaAAP6-3B, a regulator of grain protein content selected during wheat improvement
Source: BMC Plant Biol. 2018 Apr 23;18:71. doi: 10.1186/s12870-018-1280-y (PMC5914022; doi:10.1186/s12870-018-1280-y)
Supplement: Supplementary file 3 — Table S1.TaAAP6-3B genotypes and Locus polymorphism in 115 wheat lines. (DOCX 31 kb) [file 12870_2018_1280_MOESM3_ESM.docx]

**Table S1**

| **No.** | **Accession** | **cultivar groups** | **Sub^a^** | **Polymorphic SNPs** | | | | **Loci polymorphism** |
| --- | --- | --- | --- | --- | --- | --- | --- | --- |
|  |  |  |  | **wsnp_Ku_c38911_47455924** | **BS00048355_51** | **RAC875_c39926_313** | **BS00090869_51** |  |
| 1 | ZM08815 | landraces | Sub-II | AA | GG | GG | CC | Type1 |
| 2 | ZM08818 | landraces | Sub-II | AA | GG | GG | CC | Type1 |
| 3 | ZM08807 | landraces | Sub-I | AG | GG | AG | TC | Type6 |
| 4 | ZM08814 | landraces | Sub-I | AG | GG | AG | TC | Type11 |
| 5 | ZM08816 | landraces | Sub-I | AG | GG | AG | TC | Type6 |
| 6 | ZM08820 | landraces | Sub-I | AG | GG | AG | TC | Type6 |
| 7 | ZM08824 | landraces | Sub-I | AG | GG | AG | TC | Type6 |
| 8 | ZM11506 | landraces | Sub-I | AG | GG | AG | TC | Type11 |
| 9 | ZM11532 | landraces | Sub-I | AG | AG | AG | TC | Type12 |
| 10 | ZM11565 | landraces | Sub-I | AG | GG | AG | TC | Type6 |
| 11 | ZM11805 | landraces | Sub-I | AG | GG | AG | TC | Type6 |
| 12 | ZM11806 | landraces | Sub-I | AG | GG | AG | TC | Type6 |
| 13 | ZM11812 | landraces | Sub-I | AG | GG | AG | TC | Type6 |
| 14 | ZM11856 | landraces | Sub-I | AG | GG | AG | TC | Type6 |
| 15 | ZM11857 | landraces | Sub-I | AG | GG | AG | TC | Type11 |
| 16 | ZM11870 | landraces | Sub-I | AG | GG | AG | TC | Type6 |
| 17 | ZM11891 | landraces | Sub-I | AA | GG | AG | TC | Type3 |
| 18 | ZM11930 | landraces | Sub-I | AA | GG | AG | CC | Type4 |
| 19 | ZM11931 | landraces | Sub-II | AA | AG | GG | CC | Type2 |
| 20 | ZM11947 | landraces | Sub-I | AG | GG | AG | TC | Type11 |
| 21 | ZM11948 | landraces | Sub-I | AG | GG | AG | TC | Type11 |
| 22 | ZM11955 | landraces | Sub-I | AA | GG | AG | TC | Type3 |
| 23 | ZM11960 | landraces | Sub-II | AG | GG | GG | CC | Type9 |
| 24 | ZM12064 | landraces | Sub-I | AG | GG | AG | TC | Type6 |
| 25 | ZM12070 | landraces | Sub-I | AG | GG | AG | TC | Type6 |
| 26 | ZM12072 | landraces | Sub-I | AG | GG | AG | TC | Type11 |
| 27 | ZM12073 | landraces | Sub-I | AG | GG | AG | TC | Type11 |
| 28 | ZM12075 | landraces | Sub-I | AA | GG | AG | TC | Type3 |
| 29 | ZM1813 | landraces | Sub-I | AG | GG | AG | CC | Type8 |
| 30 | ZM2511 | landraces | Sub-I | AG | GG | AG | TC | Type11 |
| 31 | ZM3489 | landraces | Sub-I | AG | GG | AG | TC | Type11 |
| 32 | ZM4711 | landraces | Sub-I | AG | GG | AG | CC | Type8 |
| 33 | ZM4780 | landraces | Sub-I | AG | GG | AG | CC | Type8 |
| 34 | ZM4787 | landraces | Sub-I | AG | GG | AG | CC | Type8 |
| 35 | ZM4846 | landraces | Sub-II | AG | AG | GG | CC | Type10 |
| 36 | ZM5453 | landraces | Sub-I | AG | GG | AG | TC | Type11 |
| 37 | ZM5465 | landraces | Sub-I | AG | GG | AG | TC | Type11 |
| 38 | ZM5479 | landraces | Sub-I | AG | GG | AG | TC | Type11 |
| 39 | ZM5480 | landraces | Sub-I | AG | GG | AG | TC | Type11 |
| 40 | ZM5487 | landraces | Sub-I | AG | GG | AG | TC | Type11 |
| 41 | ZM5567 | landraces | Sub-I | AG | GG | AG | TC | Type11 |
| 42 | ZM5574 | landraces | Sub-I | AG | GG | AG | TC | Type11 |
| 43 | ZM5723 | landraces | Sub-I | AG | GG | AG | TC | Type11 |
| 44 | ZM5782 | landraces | Sub-I | AG | GG | AG | CC | Type8 |
| 45 | ZM5826 | landraces | Sub-I | AG | AG | AG | CC | Type13 |
| 46 | ZM5854 | landraces | Sub-I | AG | GG | AG | TC | Type11 |
| 47 | ZM5897 | landraces | Sub-I | AG | AG | AG | TC | Type7 |
| 48 | ZM6202 | landraces | Sub-II | AA | AG | GG | CC | Type2 |
| 49 | ZM6243 | landraces | Sub-I | AG | GG | AG | TC | Type11 |
| 50 | ZM6261 | landraces | Sub-I | AG | GG | AG | TC | Type11 |
| 51 | ZM6333 | landraces | Sub-I | AG | GG | AG | CC | Type8 |
| 52 | ZM6559 | landraces | Sub-I | AA | GG | AG | TC | Type3 |
| 53 | ZM7186 | landraces | Sub-I | AG | GG | AG | TC | Type11 |
| 54 | ZM7455 | landraces | Sub-I | AG | GG | AG | TC | Type11 |
| 55 | ZM7716 | landraces | Sub-I | AG | GG | AG | TC | Type11 |
| 56 | ZM8327 | landraces | Sub-II | AG | AG | GG | CC | Type10 |
| 57 | Bai lang mai | landraces | Sub-I | AA | GG | AG | TC | Type3 |
| 58 | Bang da dong mai | landraces | Sub-I | AG | GG | AG | TC | Type6 |
| 59 | Ben di hong mai | landraces | Sub-II | AA | AG | GG | CC | Type2 |
| 60 | Zang chun 6 hao | landraces | Sub-I | AG | GG | AG | TC | Type6 |
| 61 | Fu lan ni | landraces | Sub-II | AG | GG | GG | CC | Type9 |
| 62 | Jia cha bang da dong mai-4 | landraces | Sub-II | GG | GG | GG | CC | Type14 |
| 63 | Jia cha bang da dong mai-5 | landraces | Sub-II | AA | AG | AG | TC | Type5 |
| 64 | China spring | landraces | Sub-I | AG | GG | AG | CC | Type8 |
| 65 | 1522 | modern cultivars | Sub-II | AG | GG | GG | CC | Type9 |
| 66 | 7268 | modern cultivars | Sub-I | AG | GG | AG | TC | Type6 |
| 67 | W12R | modern cultivars | Sub-II | AA | GG | GG | CC | Type1 |
| 68 | W12S | modern cultivars | Sub-II | AA | GG | GG | CC | Type1 |
| 69 | W138 | modern cultivars | Sub-II | AA | GG | GG | CC | Type1 |
| 70 | W139 | modern cultivars | Sub-II | AG | AG | GG | CC | Type10 |
| 71 | W1718 | modern cultivars | Sub-II | AG | GG | GG | CC | Type9 |
| 72 | W29 | modern cultivars | Sub-II | AA | GG | GG | CC | Type1 |
| 73 | W47 | modern cultivars | Sub-II | AG | AG | GG | CC | Type10 |
| 74 | W5239 | modern cultivars | Sub-II | AA | GG | GG | CC | Type1 |
| 75 | WJ36 | modern cultivars | Sub-II | AA | GG | GG | CC | Type1 |
| 76 | Chuan mai28 | modern cultivars | Sub-II | AG | GG | GG | CC | Type9 |
| 77 | Chuan mai36 | modern cultivars | Sub-II | AA | GG | GG | CC | Type1 |
| 78 | Chuan mai41 | modern cultivars | Sub-II | AA | GG | GG | CC | Type1 |
| 79 | Chuan mai42 | modern cultivars | Sub-II | AA | GG | GG | CC | Type1 |
| 80 | Chuan mai44 | modern cultivars | Sub-II | AG | GG | GG | CC | Type9 |
| 81 | Chuan mai45 | modern cultivars | Sub-II | AG | AG | GG | CC | Type10 |
| 82 | Chuan mai46 | modern cultivars | Sub-I | AG | GG | GG | CC | Type9 |
| 83 | Chuan mai51 | modern cultivars | Sub-II | AA | GG | GG | CC | Type1 |
| 84 | Chuan mai54 | modern cultivars | Sub-I | AA | AG | GG | CC | Type2 |
| 85 | Chuan mai56 | modern cultivars | Sub-II | AG | GG | AG | TC | Type6 |
| 86 | Chuan nong12 | modern cultivars | Sub-I | AG | AG | AG | TC | Type12 |
| 87 | Chuan nong16 | modern cultivars | Sub-I | AG | GG | GG | CC | Type9 |
| 88 | Chuan nong17 | modern cultivars | Sub-I | AA | GG | AG | TC | Type3 |
| 89 | Chuan nong18 | modern cultivars | Sub-I | AG | AG | AG | TC | Type7 |
| 90 | Chuan nong19 | modern cultivars | Sub-I | AA | GG | AG | TC | Type3 |
| 91 | Chuan nong20 | modern cultivars | Sub-I | AG | AG | AG | TC | Type7 |
| 92 | Chuan nong21 | modern cultivars | Sub-II | AG | AG | AG | TC | Type7 |
| 93 | Chuan nong22 | modern cultivars | Sub-II | AG | AG | AG | TC | Type7 |
| 94 | Chuan nong23 | modern cultivars | Sub-II | AG | AG | AG | TC | Type12 |
| 95 | Chuan nong27 | modern cultivars | Sub-II | AG | AG | GG | CC | Type10 |
| 96 | Chuan yu12 | modern cultivars | Sub-II | AG | AG | GG | CC | Type10 |
| 97 | Chuan yu16 | modern cultivars | Sub-II | AG | AG | GG | CC | Type10 |
| 98 | Chuan yu17 | modern cultivars | Sub-II | AG | AG | GG | CC | Type10 |
| 99 | Chuan yu18 | modern cultivars | Sub-II | AG | AG | GG | CC | Type10 |
| 100 | Chuan yu19 | modern cultivars | Sub-II | AA | AG | GG | CC | Type2 |
| 101 | Chuan yu20 | modern cultivars | Sub-II | AA | AG | GG | CC | Type2 |
| 102 | Chuan yu21 | modern cultivars | Sub-II | AG | AG | GG | CC | Type10 |
| 103 | Chuan yu23 | modern cultivars | Sub-II | AA | GG | GG | CC | Type1 |
| 104 | Chuan yu24 | modern cultivars | Sub-II | AA | GG | GG | CC | Type1 |
| 105 | Mian 26 | modern cultivars | Sub-II | AA | GG | GG | CC | Type1 |
| 106 | Mian 33 | modern cultivars | Sub-II | AA | GG | GG | CC | Type1 |
| 107 | Mian 37 | modern cultivars | Sub-II | AA | GG | AG | TC | Type3 |
| 108 | Mian 45 | modern cultivars | Sub-II | AA | AG | GG | CC | Type2 |
| 109 | Mian1848 | modern cultivars | Sub-II | AA | GG | GG | CC | Type1 |
| 110 | Mian yang20 | modern cultivars | Sub-II | AA | GG | GG | CC | Type1 |
| 111 | Mian yang28 | modern cultivars | Sub-II | AA | GG | GG | CC | Type1 |
| 112 | Ri ka ze 2 | modern cultivars | Sub-I | AG | AG | AG | CC | Type13 |
| 113 | Zhen 9023 | modern cultivars | Sub-II | AA | GG | GG | CC | Type1 |
| 114 | 13F10 | modern cultivars | Sub-II | AG | GG | GG | CC | Type9 |
| 115 | Zi nuo168 | modern cultivars | Sub-II | AG | GG | GG | CC | Type9 |

Sub^a^, *TaAAP6-3B* haplotypes; Sub-I: *TaAAP6-3B-I* and Sub-II: *TaAAP6-3B-I*. 40 polymorphic SNPs of 90K Illumina iSelect array from of 3B chromosome as shown in Fig. 7; 40 types were classified in 115 Varieties.
