# Supplementary material for: TaAAP6-3B, a regulator of grain protein content selected during wheat improvement
Source: BMC Plant Biol. 2018 Apr 23;18:71. doi: 10.1186/s12870-018-1280-y (PMC5914022; doi:10.1186/s12870-018-1280-y)
Supplement: Supplementary file 6 — Table S3. Comparisons the GPC and TKW between TaAAP6-3B-I and TaAAP6-3B-II about 115 lines in five environments. (DOCX 18 kb) [file 12870_2018_1280_MOESM6_ESM.docx]

**Table S4**

| Variant region | Putative regulatory element | Putative function |
| --- | --- | --- |
| -745 to -748bp | ACGT element | required for etiolation-induced expression of erd1 (early responsive to dehydration) |
| -749 to -753bp | Sulfur-responsive element | Binding sequence of auxin response factor (ARF) |
| -897 to -900bp | YACT element | For mesophyll-specific gene expression |
| -1008 to -1014bp | SEF4 motif | interact with a soybean storage protein enhancer |
| -1474 to -1478bp | RAV1AAT | A novel DNA-binding protein, relatively high in rosette leaves and roots |
| -1547 to -1551bp | Root motif tapox1 | Evaluation in tobacco of the organ specificity and strength of the rol D promoter, domain A of the 35S promoter |

Putative *cis*-acting regulatory element predicted in the variant SNPs or InDel regions by PLACE analysis (http://www.dna.affrc.go.jp/PLACE/).
