# Supplementary material for: TaAAP6-3B, a regulator of grain protein content selected during wheat improvement
Source: BMC Plant Biol. 2018 Apr 23;18:71. doi: 10.1186/s12870-018-1280-y (PMC5914022; doi:10.1186/s12870-018-1280-y)
Supplement: Supplementary file 7 — Table S5. Contents of protein grain from genotypic classes among 197 individuals from a F2 population. (DOCX 15 kb) [file 12870_2018_1280_MOESM7_ESM.docx]

**Table S5**

| Genotype | | No.of plants | Mean±s.d (%). | Range (%) |
| --- | --- | --- | --- | --- |
| *TaAAP6-3B-I* homozygotes | 80 | | 16.13±0.01(a) | 14.2-19.99 |
| Heterozygtes | 80 | | 15.46±0.02(b) | 13.5-19.1 |
| *TaAAP6-3B-II* homozygotes | 37 | | 14.60±0.03(c) | 13.08-17.31 |

Homozygous genotypes for the ZM5453 and ZhongKemai138 alleles and the heterozygous genotype were determined by two markers *BI*and *BII*. a, b and c indicate significant differences determined by the Duncan test, *P*≤0.001.
